# Odorant-Dependent Generation of Nitric Oxide in Mammalian Olfactory Sensory Neurons

**DOI:** 10.1371/journal.pone.0005499

**Published:** 2009-05-11

**Authors:** Daniela Brunert, Stefan Kurtenbach, Sonnur Isik, Heike Benecke, Günter Gisselmann, Wolfgang Schuhmann, Hanns Hatt, Christian H. Wetzel

**Affiliations:** 1 Lehrstuhl für Zellphysiologie, Ruhr-Universität Bochum, Bochum, Germany; 2 Lehrstuhl für Analytische Chemie, AG Elektroanalytik und Sensorik, Ruhr-Universität Bochum, Bochum, Germany; 3 International Graduate School of Neuroscience, Ruhr-Universität Bochum, Bochum, Germany; Vrije Universiteit Amsterdam, Netherlands

## Abstract

The gaseous signalling molecule nitric oxide (NO) is involved in various physiological processes including regulation of blood pressure, immunocytotoxicity and neurotransmission. In the mammalian olfactory bulb (OB), NO plays a role in the formation of olfactory memory evoked by pheromones as well as conventional odorants. While NO generated by the neuronal isoform of NO synthase (nNOS) regulates neurogenesis in the olfactory epithelium, NO has not been implicated in olfactory signal transduction. We now show the expression and function of the endothelial isoform of NO synthase (eNOS) in mature olfactory sensory neurons (OSNs) of adult mice. Using NO-sensitive micro electrodes, we show that stimulation liberates NO from isolated wild-type OSNs, but not from OSNs of eNOS deficient mice. Integrated electrophysiological recordings (electro-olfactograms or EOGs) from the olfactory epithelium of these mice show that NO plays a significant role in modulating adaptation. Evidence for the presence of eNOS in mature mammalian OSNs and its involvement in odorant adaptation implicates NO as an important new element involved in olfactory signal transduction. As a diffusible messenger, NO could also have additional functions related to cross adaptation, regeneration, and maintenance of MOE homeostasis.

## Introduction

Nitric oxide (NO) is a small gaseous molecule that can diffuse through lipid membranes and plays important roles in various intra- and inter-cellular signalling processes [1]. Three major isoforms of the NO-generating enzyme NO-synthase (NOS) occur in mammalian tissues: two Ca^2+^-dependent constitutively expressed isoforms, neuronal NOS (nNOS) and endothelial NOS (eNOS), as well as an inducible isoform (iNOS). All three isoforms occur in the central olfactory system of rodents [2], but the presence and function, if any, of NOS in the peripheral olfactory system is controversial. nNOS functions in the embryonic development of the main olfactory neuroepithelium (MOE) but is down regulated shortly after birth [3]. iNOS also occurs only in the early embryonic MOE [4]. NOS isoforms, however, could not be detected in the MOE of mature rodents. Yet, despite this lack of evidence for NO-synthase in mature OSNs several studies suggested that NO potentially modulates one or more elements of olfactory signal transduction [5], [6], [7], [8], [9]. We therefore hypothesized that at least one NOS isoform, most likely eNOS, occurs in the MOE and attempted to show the presence, functionality and possible role of eNOS in the OE of adult mice.

## Results

### eNOS is expressed in mouse OSNs

**Figure 1:**
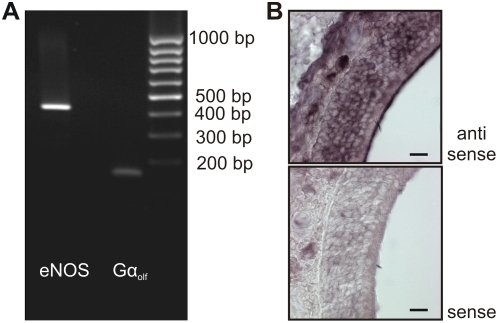
mRNA of the endothelial isoform of NO-synthase (eNOS) is expressed in olfactory sensory neurons. *A,* RT-PCR analysis of 1500 purified OSNs with primers specific for eNOS and Gα_olf_. *B,* *In situ* hybridization of eNOS-specific anti-sense and sense probes to cryosections of the murine olfactory epithelium. The scale bars represent 20 µm.

We tested the MOE of adult mice for expression of eNOS at the mRNA and protein levels. In order to obtain an enriched population of OSNs, we dissected the olfactory epithelium of homozygous OMP-GFP mice [10] expressing GFP under control of the promoter of the olfactory marker protein (OMP). In these mice, most mature OSNs are labeled by GFP-expression. Cells of the MOE were dissociated and green fluorescent neurons were purified by fluorescence-activated cell sorting (FACS). mRNA from 1500 neurons was isolated and reverse-transcribed into cDNA. PCR with primers for Gαolf, a known member of the OSN signal transduction cascade, served as a control and confirmed successful reverse transcription from the neurons. Specific primers detected amplified fragments of the expected size of 427 bp for eNOS and 100 bp for Gαolf, indicating the presence of eNOS transcripts in OSNs (Figure 1A). Since FAC-sorting can provide only up to 99% purity of the cell sample we performed *in situ* hybridization of specific riboprobes to cryosections of the murine nose. Consistent with the findings in RT-PCR, antisense probes strongly labeled the MOE (Figure 1B). Stained structures were especially prominent in the layer containing the olfactory knobs and the OSN somata, but also occurred in the upper layers of the lamina propria.

**Figure 2:**
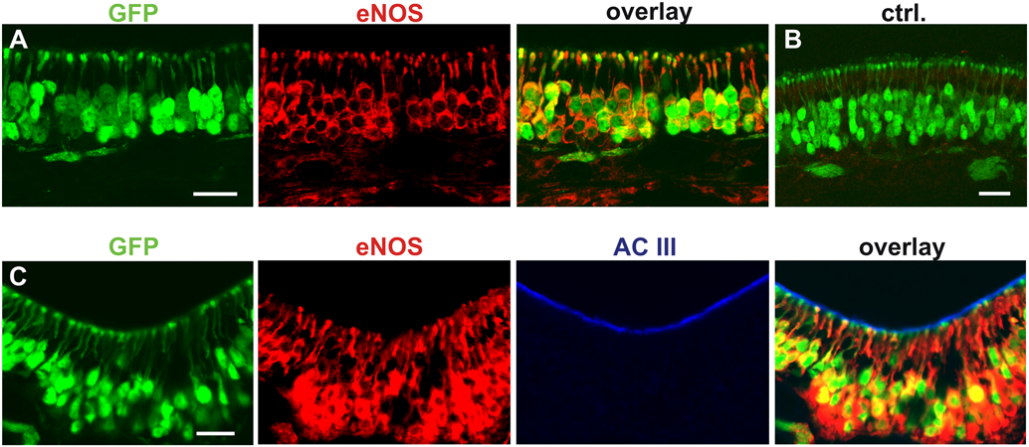
eNOS-protein is localized to somata, dendrites and olfactory knobs but not to the cilia of mature OSNs. *A,* eNOS immunostaining of cryosections of the olfactory epithelium of OMP-GFP mice. Pictures show endogenous GFP-fluorescence of OSNs (green), eNOS-specific immunostaining (red) as well as the overlay of the endogenous fluorescence and the immunostaining. *B,* Control without primary antibody. *C,* Double immunostaining of GFP-positive OSNs with antibodies for eNOS and adenylyl cyclase type III. Pictures show endogenous GFP-fluorescence of OSNs (green), eNOS-specific immunofluorescence (red), adenylyl cyclase type III (blue) and the overlay. The scale bars represent 20 µm.

In order to confirm the presence of eNOS in the adult OE, we also analysed eNOS expression in the olfactory epithelium at the protein level using immunofluorescence labelling with rabbit polyclonal anti-eNOS antibodies. The binding specificity of these antibodies was verified by immunohistochemistry on cryo sections of the OE of eNOS deficient mice (eNOSdelMu). These mice express a truncated eNOS protein lacking the NADPH binding domain of the protein that is unable to synthesize NO [11]. Immunohistochemical staining was significantly less in these sections (data not shown), although it was not completely absent because the antibody can bind to the truncated protein. eNOS-specific fluorescent signals were detected in what appeared to be all OSNs of OMP-GFP mice, indicated by co-fluorescence with GFP. In contrast, antibodies against nNOS showed no positive cells in the mature MOE as reported previously [3] (data not shown). The immunofluorescence occurred in the OSN somata, dendrites and knobs (Figure 2A and B), but not in the cilia. Double immunofluorescence labeling of eNOS and adenylyl cyclase type 3 (ACIII) [12], a protein of the canonical olfactory signal transduction cascade known to localize primarily in the ciliary compartment of OSNs, confirmed that olfactory cilia did not show any detectable eNOS staining (Figure 2C).

### eNOS synthesizes NO in isolated OSNs in response to odorant stimulation

**Figure 3:**
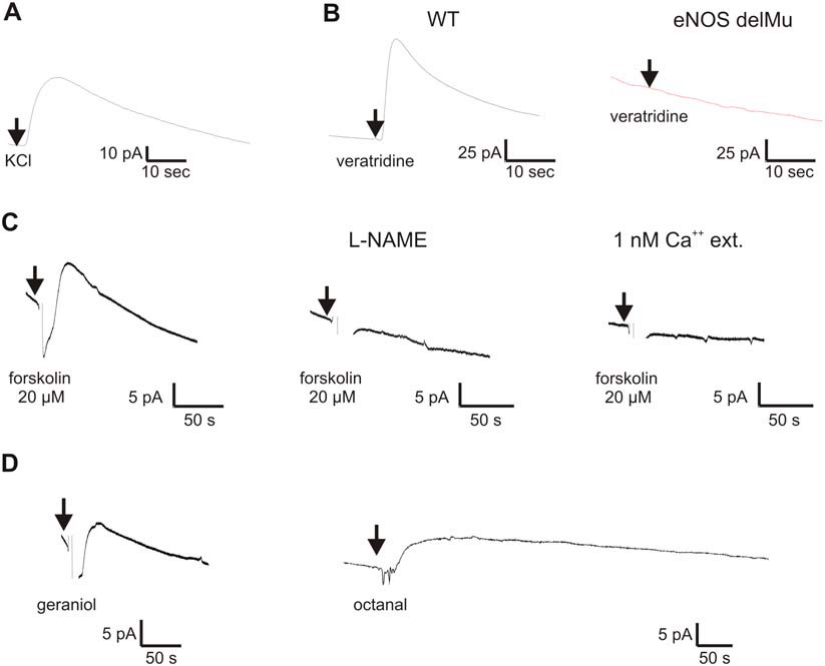
Amperometric NO-recordings of individual OSNs of wild-type, OMP-GFP, or eNOS-deficient mutant mice. *A,* Application of KCl (45 mM) induces a robust NO-signal in an isolated wild-type OSNs. *B,* Veratridine (10 µM) induces a NO-signal in a wild-type OSN, but fails to induce NO release in an OSN of an eNOS-deficient mutant mouse (eNOS-delMu). *C,* Stimulation of the olfactory signal transduction pathway with forskolin (20 µM) led to a clear NO-signal in OSNs of OMP-GFP mice. Signals could be prevented by pre-incubation with L-NAME (1 mM) or by performing the experiments in extracellular medium with low Ca^2+^-concentration (1 nM). *D,* Application of the odorants geraniol (200 µM) or octanal (500 µM) produced NO-signals in OMP-GFP mice. Arrows mark the application of the indicated stimulus. Large deflections of the signal at the time of application represented artefacts that occurred in some experiments (forskolin, geraniol). These artefacts were blanked for clarity.

We next examined whether NO can be released from OSNs in a stimulus-dependent manner by NO-amperometry. Utilizing custom made NO-sensitive micro electrodes with a 10 µm diameter catalytical surface, we were capable of measuring NO-release selectively from individual isolated cells [13]. KCl-induced depolarization elicited detectable release of NO from OSNs of OMP-GFP mice, yielding currents up to 50 pA in 11 out of 20 neurons (Figure 3A), arguing the enzyme was functional.

To investigate whether the OSN-derived NO is synthesized by eNOS, we compared NO-release from OSNs of wild-type and eNOS-deficient mice. The cells were stimulated with veratridine (10 µM), a drug known to preferentially bind to activated sodium channels causing their persistent activation and producing strong membrane depolarization [14]. Veratridine elicited detectable NO-release in 10 of 10 OSNs derived from wild-type mice (2–118 pA), but in none of 8 OSNs from eNOS deficient animals (Figure 3B), arguing that exclusively eNOS is responsible for activity-dependent release of nitric oxide from murine OSNs.

To determine whether NO-release can be triggered by activation of the cyclic nucleotide signalling cascade, we examined NO-release from OSNs following application of the adenylyl cyclase activator forskolin (Figure 3C). Forskolin evoked detectable signals from 21.4% of all tested cells from wild-type mice (n = 28 cells) though from none of the cells derived from eNOS-deficient animals (n = 96 cells, data not shown). In wild-type OSNs, forskolin induced NO-signals in a dose-dependent manner with 20 µM forskolin eliciting currents from 2–13 pA (n = 6 responsive cells, mean 8.3±1.8 pA), whereas 200 µM forskolin induced currents from 8–30 pA (n = 5 responsive cells, mean amplitude 19.8±4.4 pA). Control application of up to 2% DMSO did not elicit NO-signals from OSNs. Pre-incubation with the unspecific NOS-inhibitor L-NAME (N(G)-nitro-L-arginine-methyl-ester) prevented forskolin-induced NO-release in all cells tested (n = 16 cells), arguing that eNOS can be activated through the cyclic nucleotide signalling pathway in these cells.

Cytosolic Ca^2+^ is known to regulate eNOS activity via binding of Ca^2+^-loaded calmodulin to a site located between the COOH-terminal reductase and NH_2_-terminal oxygenase domain [15]. Therefore we tested for the Ca^2+^ dependency of stimulus-induced NO-release. Stimulation of OSNs with forskolin (20 µM) in extracellular medium containing low Ca^2+^ (1 nM) failed to generate any detectable NO-release (n = 17 cells), arguing for dependency on extracellular Ca^2+^.

To investigate whether normal odorant activation would stimulate NO-release, we stimulated OSNs with the odorants geraniol (200 µM) and octanal (500 µM) (Figure 3D). None of the tested OSNs from eNOS-deficient mice showed NO-release in response to the odorants (n = 96 for both odorants, data not shown) but stimulation of wild-type neurons with geraniol led to a detectable NO-response in 3 out of 43 cells with a maximal amplitude of 10 pA while stimulation with octanal induced NO-signals in 2 out of 10 cells with a maximal amplitude of 6 pA. As neither single odorant would be expected to activate more than a few cells in the sample population, these findings argue that normal odorant activation can trigger NO release.

### eNOS deficient mice show no difference in regeneration of the MOE but differ in adaptation kinetics

**Figure 4:**
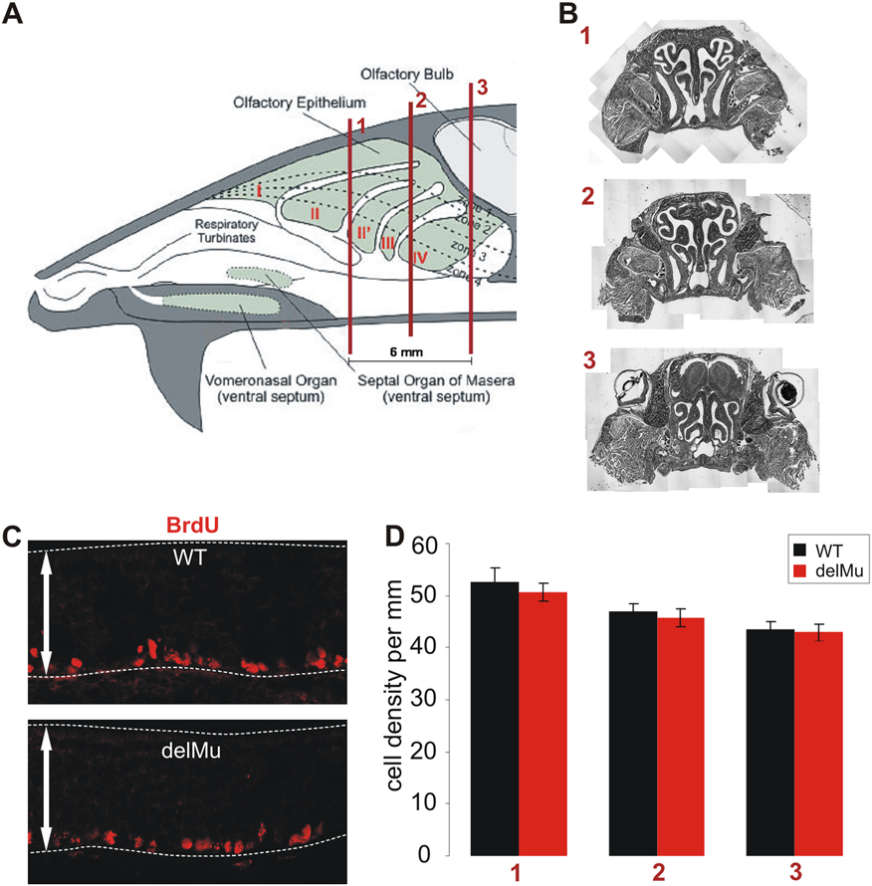
Quantification of BrdU-labeled basal cells in the olfactory epithelium of wild-type and eNOS deficient mice shows no difference in numbers of proliferating cells. (A) Schematic drawing of a mouse head with the three coronal section levels (referred to as 1, 2 and 3) that were used for quantification of BrdU positive cells. Scheme adapted from A. Puche (www.apuche.org). (B) Reconstructions of coronal sections from these section levels and their respective localization (1, 2 and 3). (C) Exemplary anti-BrdU immunofluorescence from the nasal septum of wild-type (WT) and deletion mutant (delMu) mice. Dashed lines indicate the olfactory epithelium. Arrows represent 100 µm. (D) Cell densities of BrdU positive cells of the nasal septum of WT and delMu animals for all three section levels. Error bars indicate SEM.

Given that nNOS mediates MOE development in neonatal mice but its expression is down regulated shortly after birth [3], we examined whether eNOS in the adult MOE could possibly take over the function of nNOS, perhaps regulating the lifelong regeneration of OSNs. To investigate a possible effect of eNOS derived NO on proliferation in the MOE, we BrdU-labelled wild-type and eNOS deficient mice and quantified cell densities of BrdU-positive newly generated basal cells in the septal MOE of both strains. No significant differences in the number of BrdU-positive basal cells were found in the OE of wild-type and eNOS deficient mice, arguing that eNOS has no primary function in basal cell proliferation of the mature MOE (Figure 4).

**Figure 5:**
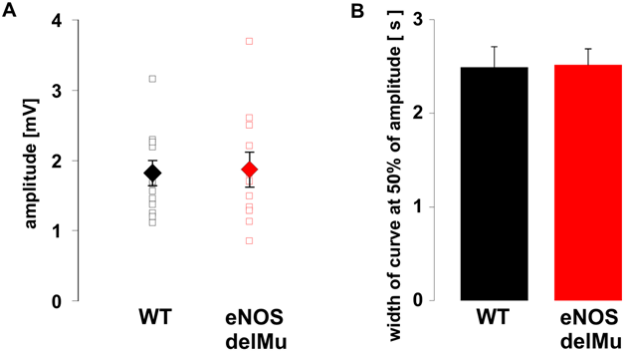
Wild-type and eNOS-deletion mutant mice show no differences in amplitude and in decay kinetics of responses to single pulses of geraniol (1 s duration). (A) Absolute (open squares) and mean amplitudes (filled diamonds) of geraniol responses. Mean values were 1.82±0.17 mV for wild-type and 1.87±0.27 mV for eNOS deletion mutants. (B) Decay kinetics measured as curve width at 50% of the response amplitude. Mean values were 2.49±0.13 s for wild-type and 2.52±0.19 s for eNOS deletion mutants. Error bars indicate SEM.

**Figure 6:**
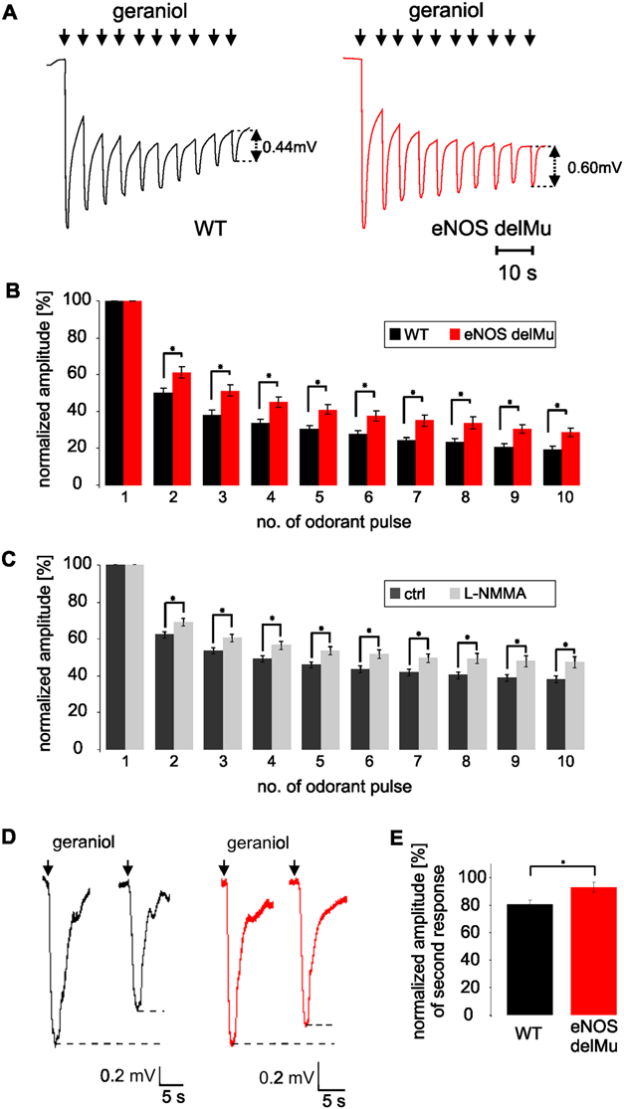
Geraniol-evoked electro-olfactogram recordings from olfactory epithelia show significant differences in adaptation for wild-type compared to eNOS-deficient mice. *A,* Representative recordings of odor-responses to repetitive application of geraniol (1 second duration; 4 seconds interstimulus interval) in wild-type (black) and eNOS deficient mice (red). *B,* Histogram of mean amplitudes of consecutive responses normalized to the amplitude of the first geraniol response in a stimulus series of wild-type (black bars) and eNOS deficient mice (red). *C*, Histogram of mean amplitudes of consecutive responses normalized to the amplitude of the first geraniol response in a stimulus series of wild-type olfactory epithelium incubated with Ringer's solution (dark grey bars) or L-NMMA (100 µM, light grey bars). Please notice that incubation of the OE with Ringer̀s solution or drug in general led to slower adaptation kinetics, but comparison of the two conditions still shows a significant difference. *D,* Representative recordings of odor responses to geraniol pulses (2 seconds duration; 28 seconds interstimulus interval). *E,* Mean amplitude of the second geraniol response normalized to the amplitude of the first response.

In order to investigate a possible functional role of eNOS in olfactory signal transduction, we recorded EOGs on wild-type and eNOS deficient mice. OSNs were stimulated with geraniol, an excitatory odorant that elicits EOG responses in a reproducible manner and possesses a very low trigeminal component [16]. Single odorant pulses (1 s) elicited EOG responses in eNOS deficient animals that did not differ in amplitude or in decay kinetics from wild-type mice (Figure 5). However, repetitive odorant stimulation showed altered adaptation in the eNOS deficient mice. The decline in the normalized signal amplitude of consecutive odorant responses (1 s @ 5s) in the eNOS deficient mice was significantly reduced compared to the decline in wild-type mice (Figure 6A and B). Reduction ranged from 9.4 to 13.2%, (n = 11 wild-type, 10 eNOS deficient mice; p = 0.009 to 0.03 for comparison of single odor pulses).

Similarly, significantly reduced response adaptation to repetitive stimuli in wild-type MOE was observed after incubation of the OE with the potent NOS inhibitor NG-monomethyl-*L*-arginine (L-NMMA, 100 mM, 15 min). Reduction of response amplitudes ranged from 6.9 to 9.2% (n = 8 control or treated animals; p = 0.015 to 0.026 for comparison of single odor pulses) (Figure 6C).

To test if the phenotype of diminished adaptation in eNOS-deficient mice is also detectable with longer interstimulus intervals we stimulated the OE with two, longer odorant pulses (2 s duration) separated by a long interstimulus interval (28 s). The second normalized mean amplitude was 92.9±3.5% of the first amplitude in eNOS deficient mice compared to 80.7±2.9% wild-type mice (n = 7 for both mouse strains, p = 0.01) (Figure 6D and E).

In conclusion our data point to NO being produced in OSNs by eNOS in a stimulus-dependent manner. This seems to enhance odor response adaptation leading to a significantly stronger adaptation than without NO.

## Discussion

While eNOS is expressed in central neurons of the olfactory system [17], we present the first evidence for its presence, functionality and possible physiological role in the MOE. Co-labelling of eNOS with OMP promoter-driven GFP expression in the layer of the mature olfactory sensory neurons argues for eNOS being expressed in mature olfactory receptor neurons, since OMP selectively labels mature [18] OSNs. The significance of finding eNOS in the layer of young olfactory neurons and basal cells is less clear. In our studies we found no evidence for a role of eNOS in basal cell proliferation though we cannot rule out functions for eNOS in cell homeostasis or migration. Its presence in the upper layer of the lamina propria can be explained by the presence of epithelial blood vessels in the lamina and the association of eNOS with blood vessels [19].

Though eNOS expression cannot be excluded completely, immunohistochemistry does not show detectable levels of eNOS protein in the olfactory cilia of mature OSNs. However, the diffusible character of NO does not necessarily require the enzyme to occur in the transduction zone in order for it to play a role in signal transduction per se. Presumably, NO could diffuse from the dendritic knob to at least the basal regions of the cilia. This would be consistent with our functional data discussed below suggesting that NO might play a role in odorant adaptation. Nonetheless, the presence of eNOS in the dendrite and soma would allow NO to additionally fulfill one or more other secondary signalling functions.

We assume the amperometric signal was specific for NO since the specificity of the nickel tretrasulfonated phthalocyanine based platinum electrodes we used has been extensively tested [20], [21]. At the potential applied, it is NO that is almost exclusively oxidized at the catalytic surface of the electrode. Additionally the NO-microsensors are coated with a Nafion membrane as an anti-interference layer which repels negatively charged molecules such as NO^2−^, the main contaminant in NO-measurements. The only remaining potential contaminant would be dopamine, which is known to be released from free nerve endings in the olfactory mucosa [22]. However, since we are measuring NO release from dissociated OSNs, dopamine contamination from free nerve endings could not interfere with the signal. Our measurements do not allow quantifying the amount and kinetics of NO release because the distance between the cell and the electrode could not be determined. As NO is not stable and gets oxidized at the electrode in close proximity to the cell, only the flux of molecules is relevant information. The concentration cannot be calculated due to the unknown volume (defined in large part by the unknown distance between cell and electrode) and the changes in flux over time. Consequently, we opted to express the flux of NO released from the cell as the current generated by oxidation of NO to NO^+^ at the sensor surface.

NO appears to be released from mature, functional OSNs. Most experiments were performed on OSNs isolated from OMP-GFP mice, which allowed using the green fluorescence of the cells in addition to the morphology to ensure the cells were mature, intact OSNs. We demonstrate that depolarization of these cells leads to NO-release and that this release is dependent on the presence of functional endothelial NO-synthase. We could also show that NO is released upon stimulation with forskolin as well as odorants, the normal physiological stimulus for OSNs. Since we also showed that NO-release is dependent on extracellular calcium, eNOS is potentially activated by the rise in intracellular calcium that occurs when the olfactory neuron is excited. Together, this argues that activation of eNOS is a general process in mature mammalian OSNs that is triggered by Ca^2+^ influx into the cells through the odorant-activated cyclic nucleotide signaling cascade as well as through opening of voltage activated Ca^2+^ channels. While simultaneous measurement of NO release and the odorant-evoked electrophysiological or calcium response of a cell would provide more direct evidence that NO is released from mature, functional OSNs, this experiment is not possible to achieve with the technology we used.

Since 100% of all tested wild-type neurons respond to veratridine with a detectable NO-signal, we suggest that most if not all OSNs are able to produce NO, not just a small subpopulation of the cells. The lower incidence in NO-release upon stimulation with KCl or forskolin does not argue against that contention, but rather more likely reflects experimentally-imposed limitations in those experiments. Similar lower activation rates in response to KCl or forskolin stimulation, for instance, were obtained in patch-clamp experiments using OSNs from OMP-GFP animals (data not shown).

The difference in EOG amplitudes between wild type and eNOS-deficient mice argues that eNOS contributes to response adaptation. The decline in amplitude of the EOG in the eNOS-deleted mutants in response to repeated odorant stimulation was significantly slower than the decline observed in wild-type mice. The fact that similar results could be obtained by blocking eNOS in the OE by the potent NOS-inhibitor L-NMMA clearly indicates that this change in adaptation is due to the deficiency of eNOS rather than some other unknown genetic factor.

Our experiments do not address the mechanism by which NO regulates adaptation. Since the major target for NO is soluble guanylyl cyclase (sGC), NO could act via sGC by increasing intracellular cGMP. Consistent with this notion, odorants can elevate cGMP [5], [23], and the rise in cGMP-concentration can be inhibited by the NOS-inhibitor L-NOARG (L-NG-nitro arginine) as well as by the NO-scavenger hemoglobin [5]. Another possible pathway is S-nitrosylation of olfactory cyclic nucleotide-gated channels [6], which has been shown to modulate the open probability of the channel. The nature of the change in adaptation kinetics as well as the longevity of this effect would be more consistent with cGMP mediation, especially, since cGMP has been implicated in a form of long lasting odor adaptation [24] that occurs upon strong odorant stimulation of OSNs and can last up to 6.5 min. Previously, this pathway has been linked to cGMP production by another gaseous messenger, carbon monoxide, produced by heme oxygenase in OSNs [25].

Collectively, our evidence for the presence of eNOS in mature mammalian OSNs and its involvement in odorant adaptation implicates NO as an important new element involved in olfactory signal transduction. Because NO is a gaseous second messenger, it could also have paracrine effects. NO has been shown to act simultaneously in both an autocrine and a paracrine manner in other cells [26]. It may therefore also subserve a paracrine function, for example mediating cross-talk with other cells in the MOE such as sustentacular cells, glands, and basal cells, or even neighbouring OSNs. Establishing the presence of eNOS in the MOE therefore may not only further our understanding of olfactory signal transduction but also shed new light on research on cross adaptation to different odorants, regeneration, and maintenance of MOE homeostasis.

## Materials and Methods

### Animals

For experiments wild-type (C57BL6) and OMP-GFP mice [10] (provided by P. Mombaerts, Max Planck Institute of Biophysics, Frankfurt), as well as eNOS deficient mice (eNOS deletion mutant (eNOS delMu); genetic background C57BL6, purchased from A. Gödecke [11]) were used. Mice were caged with water and commercial food available *ad libidum*. All animal experiments were carried out in accordance with the European Union Community Council guidelines.

### Immunohistochemistry

Adult mice (6 to 8 weeks) were sacrificed by CO_2_ and decapitated. Heads were fixed in 4% paraformaldehyde/PBS for 1 hour, decalcified in 0.5 M EDTA-solution for 4 days and cryoprotected in 30% sucrose/PBS. Coronal cryosections of the nose (12 µm) were obtained using a Leica CM3050S cryomicrotome. For fluorescence staining sections were incubated with 10% normal goat serum diluted in PBS^−−^ with 0.1% Triton-X 100 to block unspecific binding sites. Incubation of the sections with the primary antibody directed against eNOS (polyclonal rabbit anti-eNOS, Biomol, Germany) was performed over night at 4°C. Sections were incubated with secondary antibody (Alexa Goat anti-Rabbit 543 nm) for 45 min at room temperature. For colabeling of eNOS and ACIII the secondary antibody reaction was followed by incubation of the sections with anti- ACIII (Santa Cruz Biotechnologies) fused to Alexa DY631 (kindly provided by Stylianos Michalakis) for 4 hours at room temperature. Analysis of fluorescence images was performed with a confocal microscope (LSM510 Meta; Zeiss, Jena, Germany) using a 40×, 1.4 numerical aperture objective (pinhole set to one Airy unit).

### In-situ Hybridization (ISH)

Sense and antisense RNA probes were labelled with digoxigenin-UTP (Roche, Mannheim, Germany) by *in-vitro* transcription from the linearized plasmid pcDNA3haeNOS (kindly provided by A. Li, Johannes Gutenberg Universität, Mainz, Germany) containing the open reading frame of eNOS, using T7 or SP6 RNA-polymerase (Roche, Mannheim, Germany). ISH was performed on cryosections of the murine MOE according to standard methods [27]. Hybridization was visualized by incubating the slides with alkaline phosphatase-coupled anti-digoxigenin antibodies (Roche, Mannheim, Germany) and subsequent dye development with nitroblue tetrazolium and 5-bromo-4-chloro-3-indolyl phosphate.

### Preparation of olfactory sensory neurons

Adult mice (6 to 8 weeks) were sacrificed with CO_2_ and decapitated. The MOE from the septal bone and turbinates was dissected and incubated in divalent cation-free solution (140 mM NaCl, 10 mM HEPES, 10 mM glucose, pH 7.4) for 10 min at room temperature. After transferring the olfactory tissue to Ringer's solution (140 mM NaCl, 5 mM KCl, 2 mM CaCl_2_, 2 mM MgCl_2_, 10 mM HEPES, 10 mM glucose, pH 7.4) the tissue was gently triturated with fire polished Pasteur pipettes to obtain acutely dissociated OSNs. Cells were subsequently filtered through a 70 µm nylon cell strainer (Becton Dickinson) and divided into up to 5 aliquots (to allow replicate measurements) before plating them on Concanavalin A coated 35 mm Petri dishes.

### RT-PCR

The olfactory epithelium of OMP-GFP mice was dissected as described previously. The cell suspension was filtered through a 40 µm nylon cell strainer (Becton Dickinson) and cell pellets were resolved in PBS^−−^ after centrifugation at 13,000 rpm for 10 min. Cell sorting was performed in a BD FACS Vantage SE Cell Sorter (Dept. of Experimental Pneumology, Bergmannsheil, Bochum, Germany) set for highest purity. Cells were transferred to lysis buffer directly after sorting and total RNA was isolated using the RNAeasy Microkit (Qiagen, Hilden, Germany) according to the manufacturer's protocol. Subsequent to DNaseI (Invitrogen, Karlsruhe, Germany) digestion, reverse transcription of RNA obtained from about 1500 OSNs was done using 20 units of MMLV reverse transcriptase (Fermentas) and oligo (dT12-18) primer. PCR was performed in a TGradient-Cycler (Biometra, Göttingen, Germany). Gene-specific primer sets were:

G_olf_ fw 5′- CGGCCACGGGTGATGGCAAACATTACTGCTACC-3′

G_olf_ rv 5′- GCCTCTAGATCACAAGAGTTCGTACTGCTTGAG -3′   100 bp

eNOS fw 5′- GGGGCAGGCATCACCAGGAAGAAG-3′

eNOS rv 5′- TGCGCCGCCAAGAGGATACCAGT-3′       427 bp

### NO-selective micro amperometry

Custom-made NO-selective microsensors were prepared as described before [13]. The catalytic surface of the electrodes consisted of a layer of Nickel tetrasulfonate phthalocyanine tetrasodium salt (Ni-TSPc) polymerized on the microelectrode surface, resulting in a disc-like surface with a diameter of 10 µm. The electrodes were coated with a permselective nafion membrane to prevent negative ions from binding to the catalytical surface. Dissociated cells of the MOE were plated on coverslips and placed into a recording chamber with the electrodes positioned above OSNs under optical control on an inverted microscope (Axiovert 25C, Zeiss, Jena, Germany). For experiments with low extracellular calcium the cells of the MOE were washed with a solution containing low calcium buffered to 1 nM (140 mM NaCl, 5 mM KCl, 0,1 mM CaCl_2_, 10 mM EGTA, 10 mM HEPES, 10 mM glucose, pH 7.4) prior to stimulation with forskolin dissolved in the same solution. In experiments with the unspecific NOS inhibitor L-NAME (N(G)-Nitro-L-Arginine-methyl-ester, obtained from SIGMA, Deisenhofen, Germany) cells were incubated with 1 mM L-NAME at least 15 min prior to stimulation. Veratridine, forskolin (10 mM stock solution in DMSO), KCl, octanal and geraniol (1:1 diluted in DMSO) (all obtained from SIGMA, Deisenhofen, Germany) were dissolved in Ringer's solution and delivered directly to the bath as a single bolus with a stimulus pipette. The application method produced NO signals that were often preceded by large current deflections with a much faster time constant than NO-elicited electrode currents. These current deflections were considered as artifacts since they also occurred on application of Ringer's solution and/or when cells were absent. They were subsequently removed for clarity. Changes in NO concentration were measured by amperometric recording, using a low-noise Petite Ampere potentiostat (BAS Instruments, West Lafayette, USA) at a constant electrode potential of +750 mV versus an Ag/AgCl bath electrode. Data were recorded with standard data acquisition software. All results are given as the mean±SEM.

### BrdU staining

To investigate proliferation in the MOE, wild-type and eNOS deleted mutant mice (3 of each group) were injected with 50 mg BrdU per kg bodyweight. 24 hours after injection the animals were anesthetized and perfused with PFA (4%). Cryosections of the nose were obtained as described above. Sections were incubated in SSC/formamide (50%–50%) for 3 hr at 60°C. The sections were washed consecutively in SSC, PBS^−−^ and borate buffer for 5 minutes respectively and then incubated in 2 M HCl for 1 hour at 40°C. Immunohistochemical staining using mouse monoclonal anti-BrdU (Amersham Life Science, RPN 202) was performed as described before. Quantification of BrdU-positive cells was done at three levels along the anterior posterior axis of the MOE located about 3 mm apart from each other and referred to as front, middle and back. The nasal septum was chosen for quantification because it occurs and is easily recognizable in all sections and does not differ significantly from the turbinates in the number of proliferating basal cells [28]. Cell densities were calculated as number of cells per mm. All results are given as mean values±SEM.

### EOG recording

Mice were sacrificed by cervical dislocation and the nasal cavity was exposed by cutting the skull parasaggitally to the nasal septum. The septal bone with its overlying intact olfactory epithelium was used for EOG recordings as described previously [29]. For experiments that required the incubation of the OE, the hollow of the nasal cavity was filled with solution (Ringer's solution for ctrl. or L-NMMA (100 µM, obtained from SIGMA, Deisenhofen, Germany) solved in Ringer's solution) and incubated for 15 min at 37°C and removed prior to the measurements. Recording was achieved by placing an active electrode consisting of a Ringer's/agarose-filled teflon tube on the septal olfactory epithelium and connecting it with a Ag/AgCl wire to a DC amplifier (P18C, Grass Instrument Co.) referenced to an identical electrode grounding the bath. Geraniol (SIGMA, Deisenhofen, Germany) was delivered in the vapor phase using a custom made olfactometer. Odorant pulses were 1 s duration at 4 s interstimulus intervals or 2 s duration at 28 s interstimulus intervals. All results are given as mean values±SEM. Statistical significance was tested by an unpaired student's t-test.
